# Vibration Serviceability Assessment of Floor Structures: Simulation of Human–Structure–Environment Interactions Using Agent-Based Modeling

**DOI:** 10.3390/s25010126

**Published:** 2024-12-28

**Authors:** Erfan Shahabpoor, Bernard Berari, Aleksandar Pavic

**Affiliations:** 1 Department of Architecture & Civil Engineering, University of Bath, Claverton Down, Bath BA2 7AY, UK; beraribernard@gmail.com; 2 Department of Engineering, University of Exeter, Exeter EX4 4QF, UK; a.pavic@exeter.ac.uk

**Keywords:** 2DoF walking human mass-spring-damper model, crowd simulation

## Abstract

A rapidly growing body of experimental evidence in the literature shows that the effects of humans interacting with vibrating structures, other humans, and their surrounding environment can be critical for reliable estimation of structural vibrations. The Interaction-based Vibration Serviceability Assessment framework (I-VSA) was proposed by the authors in 2017 to address this, taking into account human-structure dynamic interactions (HSI) to simulate the structural vibrations experienced by each occupant/pedestrian. The I-VSA method, however, had limited provisions to simulate simultaneously multiple modes of structure in HSI, to simulate human-human and human-environment interactions, and the movement pattern of the occupants/pedestrians. This study proposes a new Agent-based Vibration Serviceability Assessment framework, termed AVSA, to address the following limitations: (a) allowing for multiple modes of structure to be simulated simultaneously, (b) to simulate effects of vibrations on gait parameters and walking pattern/routes, and (c) to simulate human-environment interactions, and movement patterns for any desired interior layout and use case. The AVSA framework was used to simulate the response and to assess the vibration serviceability of a lightweight floor under a combination of sitting and walking traffic, where three vertical modes of vibrations were engaged simultaneously. The results of the simulations show that for all tests, the experimental Cumulative Distribution Functions of the vibrations experienced by the participants are within the 95% confidence interval predicted by the AVSA method. The proposed method provides a generic and flexible framework to simulate simultaneously different interaction modalities, different human tasks and postures, and multiple modes of structure and the human body.

## 1. Introduction

Assessing the vibration serviceability of structures when human subjects are present is challenging. This is partly due to the complex nature of (a) interactions of humans with the structure, each other, and their environment (HSEI) [1,2] and (b) realistic assessment of vibration serviceability when a human is the ‘receiver’.

There is now substantial analytical and experimental evidence in the literature that suggests that ignoring HSEI can lead to significant errors in the estimated serviceability level of a structure, with substantial financial ramifications [3,4,5,6,7]. This is particularly important for structures with a high human-to-structure mass ratio and low natural frequency, such as floors, grandstands, and footbridges.

In the context of structural vibrations, several human-structure-environment interaction modalities can play critical roles in structural response.

- Human-structure dynamic interactions (HSI)
  ○ The force generated by the acceleration of the mass of the human body in response to the vibration of the structure. This force depends on the subject’s weight, task and posture, anthropometry, and the stiffness and damping of their joints [8,9,10,11,12,13].
  ○ Effects of structural vibrations on dynamic parameters of the human body, such as joint stiffness and damping [14,15].
  ○ Effects of structural vibrations on task parameters such as walking speed, pacing frequency, step length and width, and lock-in effects for a walking person [16].
- Human-environment interactions (HEI), including changes in the task, posture, and movement parameters due to environmental constraints such as changing the walking speed and direction to follow a path or to avoid obstacles [17,18,19,20].
- Human-human interactions (HHI)
  ○ Changes in task parameters, such as changes in walking speed and step length due to geometric constraints when walking in a crowd or their adaptation when walking as part of a group [18].
  ○ Changes in walking parameters as a result of visual or auditory cues such as synchronization [21,22].

Considering the inherent inter- and intra-subject variability and uncertainty of human parameters, structure parameters, and movement patterns [23], realistic simulation of such interaction modalities often requires a non-deterministic (e.g., probabilistic) framework to take these uncertainties and variabilities into account [24].

The Interaction-based Vibration Serviceability Assessment framework (I-VSA), proposed by Shahabpoor et al. [25], simulates HSI to estimate the personal vibration experience of each occupant or pedestrian. The method, however, has limited provision to simulate human-human and human-environment interactions. It requires the motion pattern, number of humans present on the structure, and their arrival and exit times as a priori. The effects of structural vibrations on dynamic parameters of the human body and task parameters are not simulated in I-VSA, either. Finally, the closed-form system of equations of motion proposed to simulate HSI does not allow for multiple modes of a structure to be simulated simultaneously.

In the past decade, significant advances have been made in vibration serviceability assessment of floor structures, including simulating the movement pattern of occupants (HHI and HEI) [19], simultaneous simulation of different tasks, i.e., walking, sitting, and standing occupants [13], and taking into account the inherent variability and uncertainty of human and structure parameters in the modeling [23]. This study builds on these important works and proposes a new Agent-based Vibration Serviceability Assessment framework (AVSA) to address the limitations of the I-VSA framework (Section 3). Compared to existing vibration serviceability assessment methods [26], the proposed AVSA framework is the first to simultaneously take into account all of the following features in a coherent, adaptable, and expandable stochastic-probabilistic framework (i.e., agent-based): (a) cross-modal simulation of the HSI, HHI, and HEI; (b) simultaneous simulation of multiple modes of structure; (c) reconfigurable and expandable simulation of human-environment interactions and movement patterns for any interior layout and use case; (d) simulating the effects of structural vibrations on dynamic parameters of the human body and task parameters; and (e) simultaneous simulation of a combination of different human postures and tasks, and autonomous switching between the tasks within the simulations.

A series of Forced Frequency Response Function (FRF) and response monitoring experiments were carried out on a lightweight floor structure to measure its occupied modal properties and vibration response, respectively, when three walking and three sitting subjects were present on the structure (Section 2). The AVSA method was then used to estimate the FRF of the occupied structure and the Cumulative Distribution Function (CDF) of the vibration experience of the occupants (Section 4.3), and the results were compared with their experimental counterparts for validation (Section 5).

## 2. Experimental Measurements

**Figure 1:**
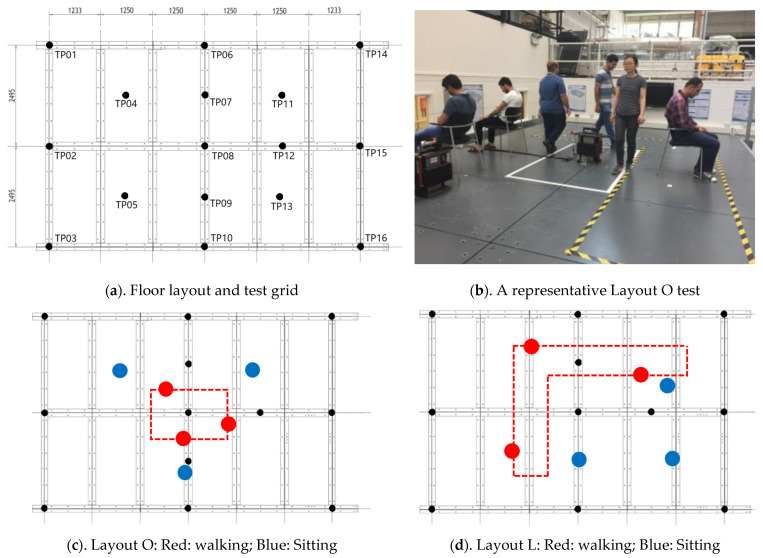
Test structure layout.

Six FRF tests and two response monitoring (RM in Table 1) tests were carried out at the Structures Laboratory of the University of Exeter on a steel floor. The test floor is comprised of twelve 2.5 m×1.25 m composite plates (a sheet of polyurethane elastomer with 19 mm thickness sandwiched between two steel plates, each with 12 mm thickness) supported by a grid of secondary and primary steel beams via bolted connections (Configuration C in Hudson and Reynolds [27]). The floor has clear dimensions of 7.5 m by 5 m (Figure 1a) and dead weight of 11,497 kg. The floor primary beams along the perimeter rest on knife edge supports, acting as roller support. The modal frequency of the first three vertical modes of vibration (Table 1) of the floor is less than 15 Hz and is deemed relevant for simulations in this study. The modal properties of the first three vertical modes of the unoccupied (empty) floor are shown in Table 1.

### 2.1. Test Setup

Six forced FRF tests, each lasting 240 s, were carried out to determine experimentally the modal properties of the empty and occupied structure. In occupied structure tests, three subjects were sitting, and three subjects were walking in two closed-loop layouts: an O-shaped layout around the mid-point of the floor targeting mainly Mode 1 of the floor, and an L-shaped layout to engage predominantly Modes 2 and 3 (Figure 1). These layouts ensure maximum HSI engagement and minimize the noise of experimental FRFs due to a steady-state closed-loop pattern. An APS Electro-Dynamics model 400 actuator [28] with 30 kg inertial mass was used to excite the structure. Chirp signals were used to excite the structure to resonance with the frequency ranges of 4–8 Hz for the first vertical mode and 12–17 Hz range for the second mode. Chirp excitation, compared with a random excitation, allows the structure to build up the resonant response more strongly and, therefore, engages HSI mechanisms more effectively. The shaker force was calculated using the acceleration measured using an Endevco model 7754-1000 piezoelectric accelerometers (PCB Piezotronics, Depew, NY, USA) attached to the actuator moving armatures. The actuator was active at TP8 (anti-node of Mode 1) for Mode 1 FRF tests and at TP13 for Modes 2 and 3 FRF tests (Figure 1a). Five male and one female healthy participants, with average age, height, and mass of 30 years, 1.69 m, and 74 kg, respectively, participated in the tests. The location time history of the walking subjects was measured using a video camera mounted above the floor.

The acceleration response of the structure was measured with a 1 kHz sampling rate using a set of 16 Honeywell QA700 and QA750 servo accelerometers (Honeywell, Charlotte, NC, USA) located at points TP1-16 on the test grid, as shown in Figure 1a. A Data Physics SignalCalc Mobilizer spectrum analyzer (Data Physics, Riverside, CA, USA) was used to generate the drive signals and to record the measured accelerations.

Further, two response monitoring tests were carried out, where the shakers were off, and the response of the structure was measured when three subjects were sitting and three subjects were walking on it (Table 1).

### 2.2. System Identification

The FRF graphs used for system identification were calculated based on the shaker force as the only excitation and the acceleration response measured at the anti-node of each mode. To minimize noise (predominantly uncorrelated extraneous excitation due to unmeasured walking forces) in each test, 12 FRF data blocks, each lasting 20 s, were recorded and averaged in the frequency domain. In each data block, the excitation lasted for the first 18 s, while the remaining 2 s allowed the response signal to die out before the acquisition of the next data block started (see [29] for more details). The number and duration of the acquisition blocks were determined based on the experimental observation that, during the walking tests, the FRFs settled after 6–8 averages and did not change noticeably with further averaging, representing the averaged effects of the quasi-stationary walking crowd on the structure over the test duration. The H1 FRF estimator was further used to reduce the effects of the unmeasured walking forces. The H1 estimator is obtained by dividing the Cross Spectral Density of the excitation response signals by the Auto Spectral Density of the excitation signal and assumes that all of the measurement noise (unmeasured walking forces in this case) is in the response signal and can be averaged out [30].

The normal pacing frequencies of the walking test subjects (measured prior to the FRF tests) were between 1.60 Hz and 1.85 Hz, with the first three harmonics at 1.60–1.85 Hz, 3.2–3.7 Hz, and 4.8–5.55 Hz, below the frequency range of the modes of interest of the structure. This resulted in the walking force spectra being relatively flat over the relevant half-power bandwidth and having considerably lower amplitudes than the shaker excitation. As a result, the walking excitation was considered as a narrow-band extraneous excitation, uncorrelated with the excitation force, the effects of which can be averaged out during experimental FRF estimation (see [17] for further details). This, however, does not mean that the effects of human walking forces are ignored in simulations. GRFs of walking subjects were applied at their corresponding locations on the structure in each time step as part of the Agent-based Model (ABM), and their effects are considered in the response simulations.

Although the location of pedestrians changed on the structure as they walked, the nominally stationary regime of the walking traffic (i.e., a constant number of walking subjects looping through the same walking route) meant that pedestrians were switching between a finite set of distinct location patterns, resulting in the FRF converging to its average value in a short span of time. The probabilistic basis of this argument is discussed extensively in [31].

Experimental FRF graphs were curve-fitted using Matlab (2021) software, and the modal frequency f [Hz], modal mass m [kg], and modal damping ratio ζ [%] of the structure were identified for each of the three vertical modes (Table 1).

**Figure 2:**
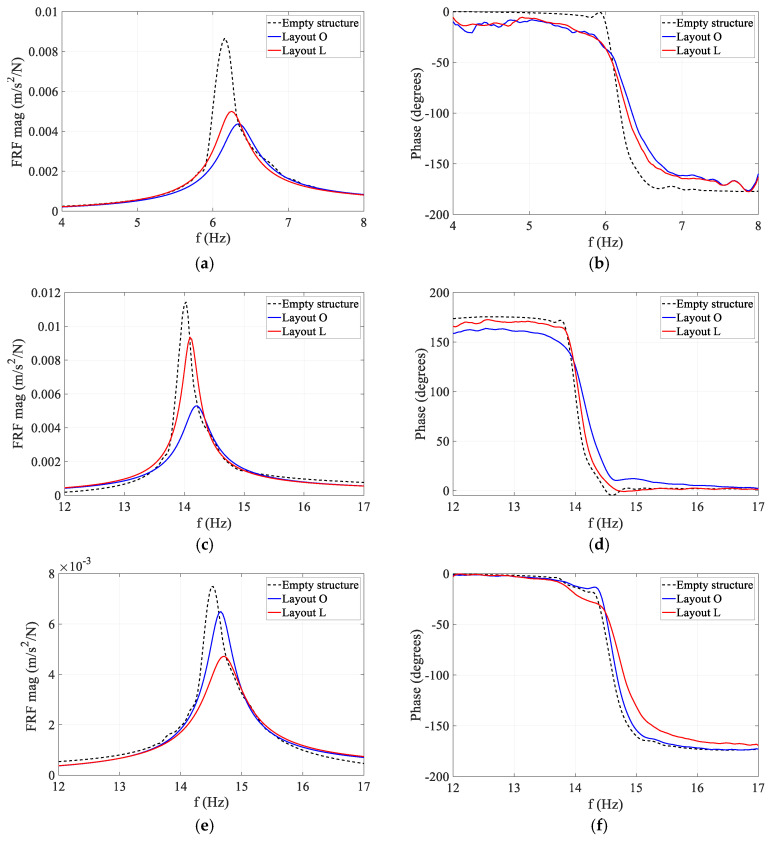
Experimental FRFs of empty and occupied structure. (**a**) Mode 1 magnitude; Reduction in peak amplitude: O: 53%; L:45%; Increase in modal frequency: O:2.8%; L:1.5%. (**b**) Mode 1 phase. (**c**) Mode 2 magnitude; Reduction in peak amplitude: O: 53%; L:23%; Increase in modal frequency: O:1.1%; L:0.6%. (**d**) Mode 2 phase. (**e**) Mode 3 magnitude; Reduction in peak amplitude: O: 9%; L:36%; Increase in modal frequency: O:0.6%; L:1.2%. (**f**) Mode 3 phase.

The magnitude and phase of the experimental accelerance FRFs of different modes are compared for Layout O and L in Figure 2. As can be seen in Figure 2, the presence of humans consistently resulted in an increase in modal frequency and modal damping ratio (reduced peak magnitude and lower phase slope) for all three vertical modes of the floor. This trend is consistent with previous observations in the literature that human occupants add damping and act as a mechanical system that changes the dynamic characteristics of the structure [8]. The consistent increase in modal frequency further highlights the fact that humans do not merely act as an added mass, in which case reduction in the modal frequency was expected instead.

Comparing the reduction percentages in FRF amplitudes shown in Figure 2, both Modes 1 and 2 are substantially affected by Layout O (53% reduction in peak), while Mode 3 was less affected (9% reduction in peak). For Layout L, however, Modes 1 (45% reduction in peak) and 3 (36% reduction in peak) are more affected than Mode 2 (23% reduction in peak). The high reduction in FRF peak of Mode 2 in Layout O and Mode 1 in Layout L is partly due to the position of sitting subjects on the floor in each layout.

## 3. Agent-Based Model

**Figure 3:**
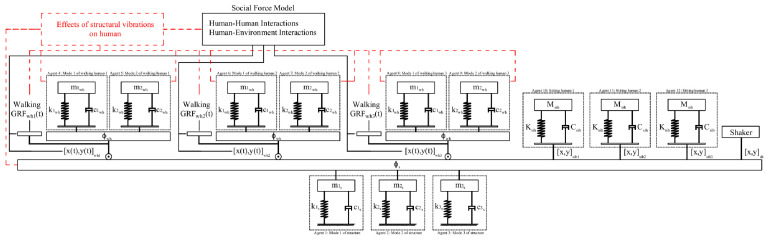
ABM architecture.

An agent-based model is developed in AVSA to simultaneously simulate the dynamic interactions of walking humans, the environment, and the structure (Figure 3). Effects of structural vibrations on human model parameters, such as joint stiffness and damping, and on walking gait parameters, such as step length and pacing frequency, are not simulated in this study. However, the proposed AVSA framework allows for simple integration of these effects in the ABM architecture where needed (Figure 3).

### 3.1. Human-Structure Dynamic Interactions

In each test, an ABM with 12 SDOF agents was modeled in MATLAB to simulate the experiment (Figure 3). Three SDOF agents represent the first three vertical modes of the floor vibrations, six SDOF agents represent three walking humans (each human represented by two agents/modes of vibration), and three SDOF agents each represent a sitting human (Figure 3).

**Figure 4:**
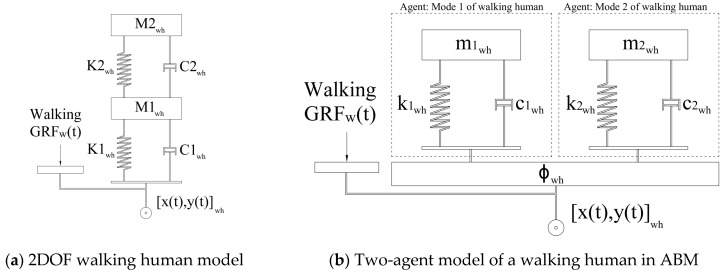

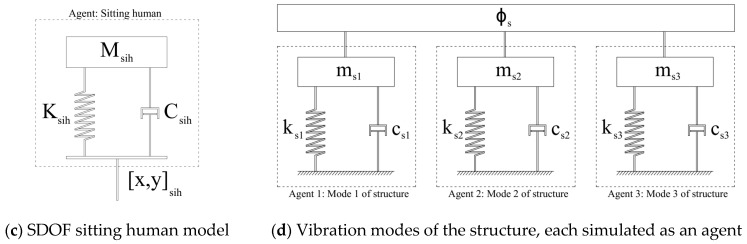
MSD models of humans and structure.

The walking human body was simulated using a linear 2DoF mass-spring-damper model (Figure 4a) with $M1_{wh}=35\;\mathrm{kg};\;M2_{wh}=40\;\mathrm{kg};\;C1_{wh}=C2_{wh}=1000\;N.s/m;\;K1_{wh}=8\times 10^{4}\;N/m,$ and $K2_{wh}=6\times 10^{4}\;N/m$ [29]. This 2DoF model results in two walking human modes of vibration with $f1_{wh}=4.3\;\mathrm{Hz}$; $\zeta 1_{wh}=0.19$; and $f2_{wh}=11.0\;\mathrm{Hz}$; $\zeta 2_{wh}=0.52$, each of which is modeled as a single-degree-of-freedom (SDOF) agent in the ABM (Figure 4b).

The acceleration of the walking human masses ($M1_{wh}$ and $M2_{wh}$) as a result of structural vibrations, generate a force (called Interaction Force $IF_{wh}\left(t\right)$ from here on) that excites the structure in a feedback loop. $IF_{wh}\left(t\right)$ is different from the walking $GRF_{w}\left(t\right)$ generated as a result of human locomotion. The force exerted on the structure by each walking individual in each time step was the sum of $IF_{wh}\left(t\right)$ and $GRF_{w}\left(t\right)$ in that timestep (Section 4).

A library of over 1000 walking GRF time histories, measured over the span of 15 years using an instrumented dual belt treadmill, was used to select $GRF_{w}\left(t\right)$ in ABM. The GRF measurements were carried out for each subject at a range of different walking speeds below and above their normal walking speed. Prior to each ABM simulation, a subject was randomly selected from the GRF dataset, and their GRF set (at different walking speeds) was assigned to each walking human model (Figure 4b). In each timestep ‘*i*’ of simulation, the instantaneous walking speed $V_{wh}\left(t_{i}\right)$ of each walking subject, determined by the ABM, was used to choose the corresponding $GRF_{w}\left(t_{i}\right)$ from this GRF set and was applied to the structure at the location of the corresponding subject (Figure 4b). The inter- and intra-subject variability of natural walking speed and pacing frequency is taken into account in the simulation through random selection of (a) walking GRFs (as explained above) and (b) the desired walking speed for each subject in the Social Force Model (SFM) (Section 3.2.1).

The dynamics of a sitting human subject were simulated using an SDOF model with $f_{sih}=6.74\;\mathrm{Hz}$; $\zeta_{sih}=0.30$; $M_{sih}=60\;\mathrm{kg};C_{sih}=1500\;N.s/m;\;$ and $K_{sih}=6\times 10^{4}\;N/m$ (Figure 4c). The SDOF model parameters were identified using the same identification methodology used for the walking human model [29]. The SDOF model of each sitting subject was modeled in the ABM as an agent located at their corresponding fixed position ${\left(x,y\right)}_{sih}$ (Figure 4c).

The first three vertical vibration modes of the structure, each represented by an SDOF agent (Figure 4d), were included simultaneously in the simulation. This multi-modal simulation is important to achieve a realistic response, particularly for structures with closely spaced modes, such as the floor structure used in tests here (Modes 2 and 3). In FRF tests, the shaker force measured during each experiment was applied to the structure at the location of the shaker (Figure 3).

### 3.2. Human-Human and Human-Environment Interactions

The movement patterns of the walking subjects on the floor as a result of their spatial interactions with each other and their environment (walls, furniture, etc.) are simulated in the ABM using SFM to find their instantaneous location, heading direction, and walking speed (Figure 4). This information was used in each timestep ‘*i*’ to specify the location of each walking subject ‘*j*’ on the structure ${\left[x\left(t_{i}\right),y\left(t_{i}\right)\right]}_{whj}\;$ and to adjust their $GRF_{whj}\left(t_{i}\right)$ (Figure 3) based on their instantaneous walking speed.

To showcase the capability of the AVSA framework for designing new structures, the model was not calibrated against experimental data, and only the input parameters available at the design stage were used in the simulations, i.e., room layout, usage scenario, and the estimated number of walking and sitting people.

#### 3.2.1. Social Force Model

In each timestep $t_{i}$, the SFM uses the mass, desired walking velocity $V_{d}$, and the position of each walking subject from the previous time step as input to calculate a resultant force $\;F_{Res}\left(t_{i}\right)$ (Equation (1)). This force is then used to determine the agent’s heading velocity $V_{a}$, and the position of each subject on the floor in the current timestep. Helbing and Molnár [32] and Carrol et al. [18] propose that $\;F_{Res}\left(t_{i}\right)$ must include (a) the motive force, $F_{M}\left(t_{i}\right)$, representing the desire of an agent to travel at a predetermined comfortable velocity (Equation (2)), (b) the inter-pedestrian force $F_{IP}\left(t_{i}\right)$, simulating the human natural desire to maintain a comfortable distance from other humans (Equation (3)), and (c) the boundary force $F_{B}\left(t_{i}\right)$, simulating the desire to move between boundaries and avoid obstacles (Equation (4)).

(1) $$F_{Res}\left(t_{i}\right)=F_{M}\left(t_{i}\right)+F_{IP}\left(t_{i}\right)+F_{B}\left(t_{i}\right)$$

(2) $$F_{M}\left(t_{i}\right)=m_{p}\frac{V_{d}-V_{a}}{t_{r}}$$

(3) $$F_{IP}\left(t_{i}\right)=c\times k_{1}e^{\frac{2r_{IP}-d_{IP}}{k_{2}}}$$

(4) $$F_{B}\left(t_{i}\right)=\{\begin{array}{c} F_{B-edge}\left(t_{i}\right)=c\times k_{1}e^{\frac{r_{B}-d_{E}}{k_{2}}}\;;\;for\;edges\\ F_{B-corner}\left(t_{i}\right)=c\times k_{1}e^{\frac{r_{B}-d_{C}}{k_{2}}}\;;\;for\;corners \end{array}$$

In Equations (1)–(4), the force constant $k_{1}=2000\;N$, distance constant $k_{2}=0.08\;m$, agent reaction time $t_{r}=0.5\;s$, mass constant $m_{p}=300\;\mathrm{kg}$, agent personal radius in inter-personal interaction $r_{IP}=0.35\;m$, and agent personal radius in relation to boundaries $r_{B}=0.25\;m$ were used in simulations. These parameters were found empirically to generate a visually convincing movement pattern and were (intentionally) not calibrated against the experimental data.

**Figure 5:**
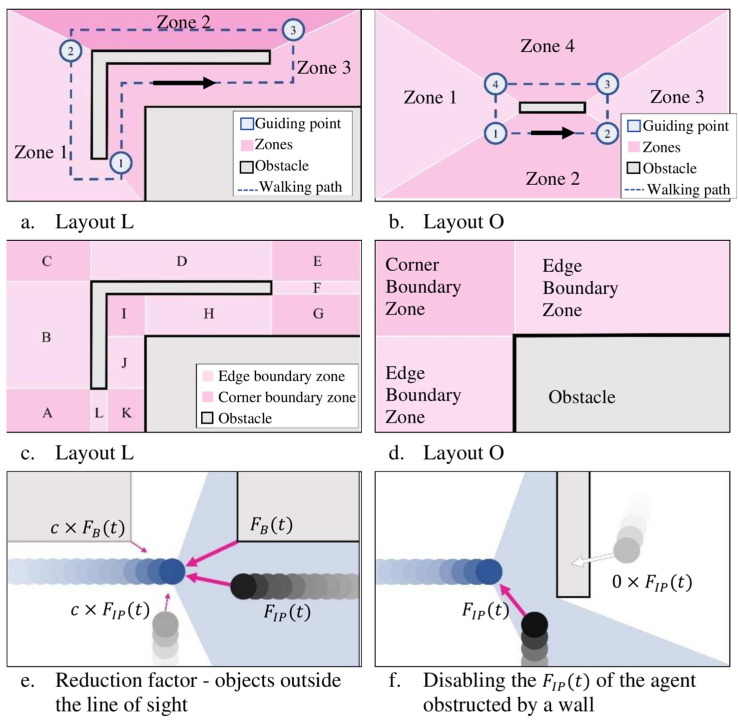
Modeling framework and factors.

In all experiments, subjects walk in loops to create a steady-state walking condition (Section 2). To simulate this pattern, the walkways in Layouts L and O are divided into a set of zones, each containing a guiding point at their exit. Within each zone, the subjects are attracted by $F_{M}\left(t_{i}\right)$ to the corresponding guiding point (Figure 5a,b). The traffic flow in both scenarios is assumed to be counterclockwise, where this assumption has no bearing on the output of the simulations.

$F_{B}\left(t_{i}\right)$ depends on the geometry of the boundary/obstacle. Walkways are divided into ‘edge’ or ‘corner’ boundary zones, and $F_{B}\left(t_{i}\right)$ is defined for each zone based on its boundary type (Figure 5c,d). Edge boundaries apply $F_{B-edge}\left(t_{i}\right)$ (Equation (4)) to the agent, where $d_{E}$ is the perpendicular distance of the agent from the edge, whereas corner boundaries apply $F_{B-corner}\left(t_{i}\right)$ (Equation (4)) to the agent, where $d_{C}$ is the total distance of the agent from the corner.

During each timestep, an agent experiences an $F_{IP}\left(t_{i}\right)$ from every other agent in the room. The force is a function of the distance between the positions of the agents. However, if some agents are obstructed by obstacles, their $F_{IP}\left(t_{i}\right)$ is zero.

Helbing and Molnár [32] underline the importance of the line of sight in pedestrians’ reactions to stimuli outside of their peripheral vision. The reduction factor c reduces the effects of stimuli outside the field of view, which this model defines as a 120° range in front of the agent (Figure 5e,f). Equations (3) and (4) use c=50% for $F_{B}\left(t_{i}\right)$ and $F_{IP}\left(t_{i}\right)$, which is calibrated empirically to achieve a realistic movement pattern without referring to the experimental data.

**Figure 6:**
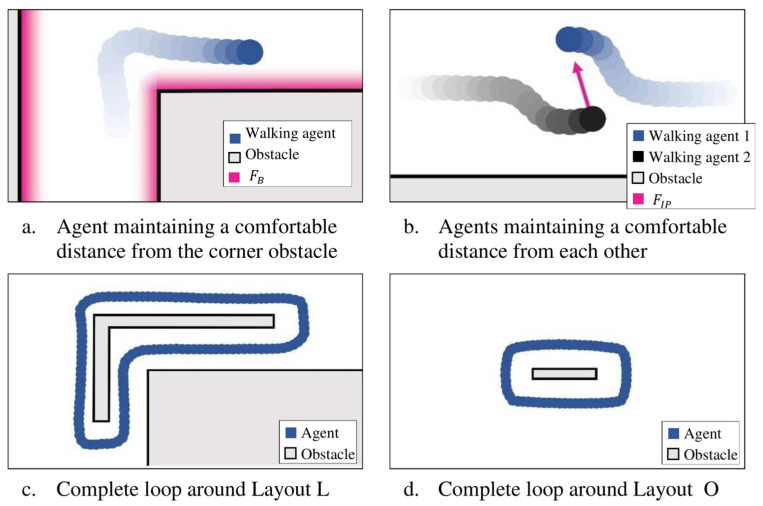
Qualitative validation of priN/Amary Social Force functions.

Figure 6a shows the spectrum of $F_{B}\left(t\right)$ and Figure 6b shows the interaction of $F_{M}\left(t\right),\;\;F_{B}\left(t\right)$ and $F_{IP}\left(t\right)$ leading to a realistic separation between an agent and boundary and between two agents while walking in the desired direction. Figure 6c,d, show a typical complete walking path for Layouts L and O, demonstrating a realistic walking pattern while avoiding obstacles and boundaries.

#### 3.2.2. SFM Quantitative Validation

The simulated movement patterns of the three walking agents for Layouts L and O are compared with the corresponding experimental data to assess the accuracy of the results. The video footage of each test is analyzed, and the time history of the location of each walking subject on the floor ${\left[x\left(t\right),y\left(t\right)\right]}_{whj}$ is extracted.

**Figure 7:**
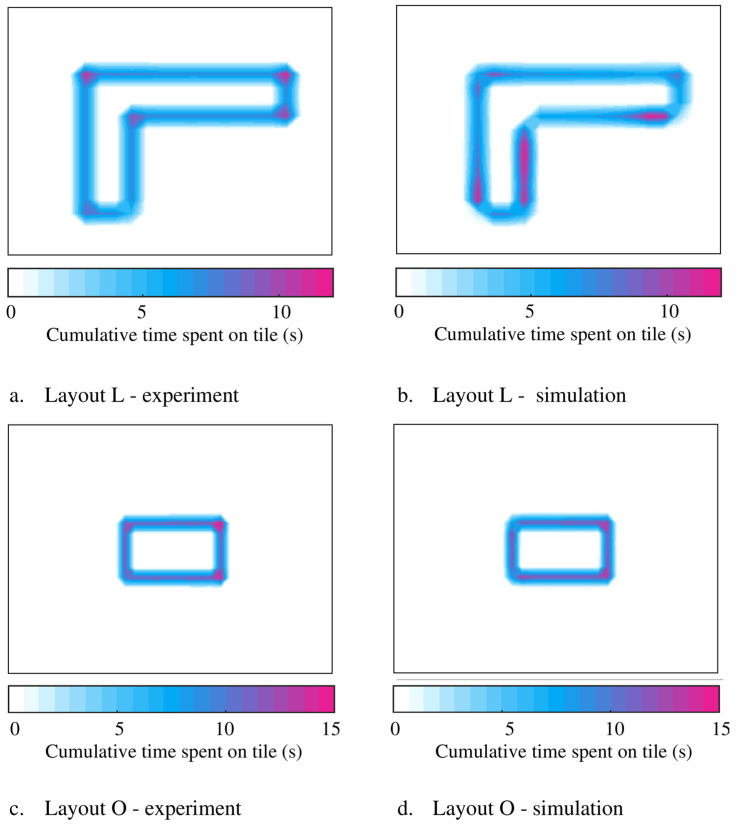
Comparisons of experimental and simulated pathway trajectories.

The floor plan is divided into a 23 × 33 grid of equal-sized tiles of approximately 22.5 cm × 22.5 cm, and the total time each agent has spent in each of the tiles is calculated and compared in Figure 7 for both analytical and experimental time histories. As can be seen in Figure 7, the analytical and experimental heat maps and the pathway trajectories are reasonably similar in shape and intensity for both Layout L and O. However, in the simulations, agents appear to round off corners and decelerate and accelerate slower around turns compared with the measurements.

**Figure 8:**
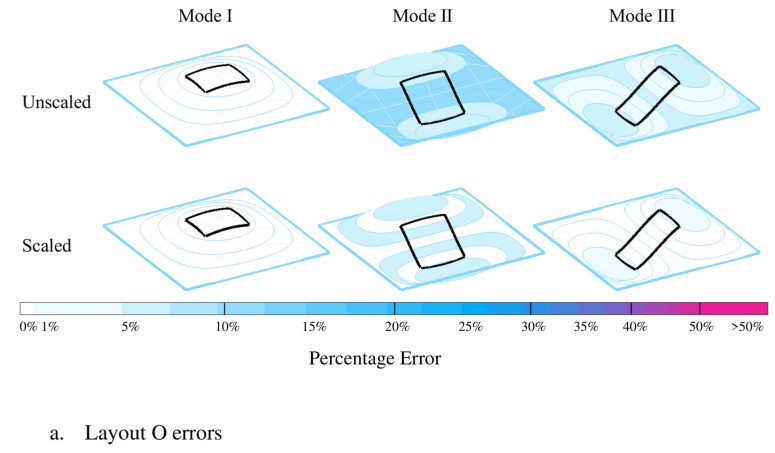

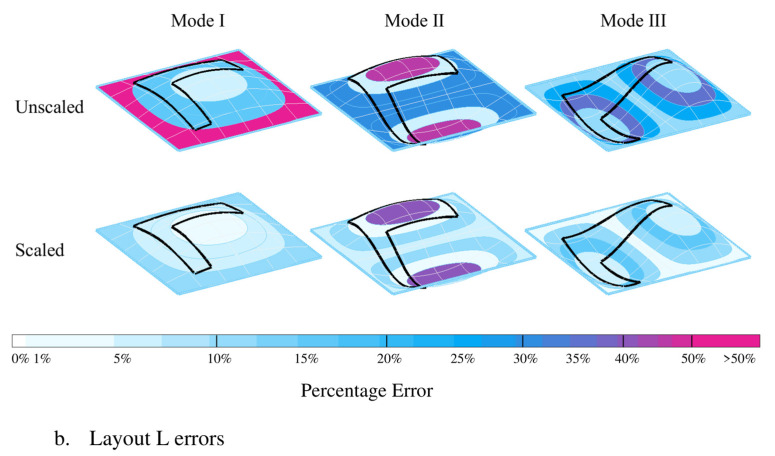
Modal errors of the simulated pathway trajectories.

To analyze the effects of walking pattern errors on structural response, for each mode of vibration, the floor plan is divided into 4 zones based on the magnitude of the corresponding unity-scaled mode shape ∅: Zone 1: 0.75<∅≤1, Zone 2: 0.5<∅≤0.75, Zone 3: 0.25<∅≤0.5, and Zone 4: 0≤∅≤0.25 (Figure 8). The total time all subjects spent in each of the zones during each test was calculated for the simulations $\left(t_{sim}\right)$ and experimental measurements $\left(t_{exp}\right)$ and the corresponding error was calculated using (Equation (5)) and shown in Figure 8 (unscaled).

(5) $$Err\left(\%\right)=\frac{\left|t_{exp}-t_{sim}\right|}{t_{exp}}\times 100$$

The error value of each zone was then scaled with the corresponding average unity-scaled mode shape magnitude for that zone (Zone 1: 0.875; Zone 2: 0.625; Zone 3: 0.375; Zone 4: 0.125) to represent their actual effects on the structural response (Figure 8—scaled). As can be seen in Figure 8, except Zone 1 of Mode 2—Layout L, all the zones/modes show scaled error values under 15%, indicating the acceptable accuracy of the simulated walking patterns. The relatively high error value of Zone 1 of Mode 2—Layout L (38%) is due to the fact that only a small part of the walking pathway passes through Zone 1, which results in low magnitudes of $t_{exp}$ and $t_{sim}$ and, consequently, high sensitivity of their corresponding errors. This, however, has no significant negative effect on the vibration response due to the small time that agents spend in Zone 1.

## 4. AVSA Framework

The AVSA framework can be divided into three stages that are carried out sequentially: Initialization, Response Simulation, and Serviceability Assessment.

### 4.1. Initialization

The first stage is to lay out the ABM structure based on the usage scenario, interior layout, and dynamic properties of the structure.

I. Modeling structural dynamics
  - Determine how many modes of structure are relevant for simulation by analyzing the modal properties of the empty structure. Use an SDOF model to simulate the contribution of each mode.
II. Modeling human dynamics
  - Determine the type/s of human posture/s or task/s relevant to the structural usage, e.g., walking, sitting, standing, etc. Assign an appropriate linear mass-spring-damper (MSD) agent model to each task/posture to simulate their dynamics. For instance, use two SDOF agents to represent the dynamics of each walking subject (assuming a 2DOF walking human model), and an SDOF agent to represent a sitting subject.
  - Assign a randomly selected (preferably measured) walking $GRF_{whj}\left(t\right)$ set to each of the walking subjects *j*. The freely available online measured walking force datasets can be used as the source.
III. Modeling human-environment interactions using SFM
  - Define the floor plan and geometry of the walkways, boundaries, obstacles, and the location of entrance and exit points.
  - Divide the walkway into a set of zones, each containing a guiding point at their exit to define the motive force $F_{M}\left(t_{i}\right)$ equation.
  - Define the ‘edge’ and ‘corner’ boundary zones and formulate the boundary force $F_{B}\left(t_{i}\right)$ equation for each zone.
  - Formulate the equation for inter-pedestrian force $F_{IP}\left(t_{i}\right)$ and the resultant force $F_{Res}\left(t_{i}\right)$. $F_{Res}\left(t_{i}\right)$ determines the heading, velocity, and position of each subject j on the floor in each time step $t_{i}$.
  - Define the set value or the statistical distribution that determines the number of human subjects entering, exiting, or present on the structure and their posture/task.

### 4.2. Response Simulation

The ABM is run through the following process in each timestep to simulate the structural response and the individualized experience of each subject:

I. Set the next time step (step ‘*i*’).
  (6) $$t_{i}=t_{i-1}+\Delta t,\;\;\;\;\Delta t=0.001\;s$$
II. SFM was run to calculate the new position of each walking subject ‘j’ on the floor ${\left[x\left(t_{i}\right),y\left(t_{i}\right)\right]}_{whj}$ and their walking speed $V_{whj}\left(t_{i}\right)$ based on their interactions with other agents and the environment.
III. Each walking person ‘j’ was moved to their new location ${\left[x\left(t_{i}\right),y\left(t_{i}\right)\right]}_{whj}$.
IV. For each walking subject ‘j’, their $V_{whj}\left(t_{i}\right)$ was used to find the walking force magnitude $GRF_{whj}\left(t_{i}\right)$ from their pre-assigned pool of GRFs.
V. The physical acceleration response of the structure from the previous timestep was transmitted to each human model (both sitting and walking) based on their location on the floor. This response is felt by each human ‘j’ as the base excitation ${\ddot{z}}_{s\to hj}\left(t_{i}\right)$ calculated using the modal acceleration response from the previous timestep (${\ddot{z}}_{sq}\left(t_{i-1}\right)$) and the mode shape ordinates at the location of human ‘j’ ($\Phi_{sq}\left(x_{j}\left(t_{i}\right),y_{j}\left(t_{i}\right)\right)$) for each mode ‘q’ of the structure:
  (7) $${\ddot{z}}_{s\to hj}\left(t_{i}\right)=\Phi_{s1}\left(x_{j}\left(t_{i}\right),y_{j}\left(t_{i}\right)\right){\ddot{z}}_{s1}\left(t_{i-1}\right)+\Phi_{s2}\left(x_{j}\left(t_{i}\right),y_{j}\left(t_{i}\right)\right){\ddot{z}}_{s2}\left(t_{i-1}\right)\;+\Phi_{s3}\left(x_{j}\left(t_{i}\right),y_{j}\left(t_{i}\right)\right){\ddot{z}}_{s3}\left(t_{i-1}\right)$$
VI. For each walking human:
  - The mode shape $\Phi_{wh}$ was used to calculate the modal base excitation experienced by mode one ${\ddot{z}}_{s\to whj,1}\left(t_{i}\right)$ and two ${\ddot{z}}_{s\to whj,2}\left(t_{i}\right)$ of the walking human.
  - The modal response of the two human modes was calculated using the Newmark integration method [30]. This was performed by taking the previous timestep displacement and velocity as initial conditions for the current step.
  - The physical acceleration response of the walking human masses $M1_{whj}$ and $M2_{whj}$ were calculated from the corresponding modal responses using $\Phi_{wh}$.
    (8) $$\left\{\begin{array}{c} {\ddot{Z}}_{whj,1}\left(t_{i}\right)\;\\ {\ddot{Z}}_{whj,2}\left(t_{i}\right)\; \end{array}\right\}=\left[\Phi_{wh}\right]\left\{\begin{array}{c} {\ddot{z}}_{whj,1}\left(t_{i}\right)\;\\ {\ddot{z}}_{whj,2}\left(t_{i}\right)\; \end{array}\right\}$$
  - The $IF_{whj}\left(t_{i}\right)$ of the 2DoF human model was calculated:
    (9) $$IF_{whj}\left(t_{i}\right)=M1_{wh}{\ddot{Z}}_{whj,1}\left(t_{i}\right)+M2_{wh}{\ddot{Z}}_{whj,2}\left(t_{i}\right)$$
VII. For each sitting human:
  - The response ${\ddot{Z}}_{sihj}\left(t_{i}\right)$ of the SDOF sitting human model to the base excitation at the location of each sitting subject ‘j’ ${\ddot{z}}_{s\to sihj}\left(t_{i}\right)$ was calculated using the Newmark integration method [30]. This was performed by taking the previous timestep displacement and velocity as initial conditions for the current step.
  - The $IF_{sihj}\left(t_{i}\right)$ of the SDOF sitting human model was calculated:
    (10) $$IF_{sihj}\left(t_{i}\right)=M_{sih}{\ddot{Z}}_{sihj}\left(t_{i}\right)$$
VIII. The total force experienced by each mode ‘q’ of the structure is calculated using $\Phi_{sq}$, taking into account $IF_{whj}\left(t_{i}\right)$ and $GRF_{whj}\left(t_{i}\right)$ of each walking subject, $IF_{sihj}\left(t_{i}\right)$ of each sitting subject, and the shaker force $F_{sh}\left(t_{i}\right)$:
  (11) $$F_{wh+sih+sh\to sq}\left(t_{i}\right)=\Phi_{sq}\left(x_{sh},y_{sh}\right)\times F_{sh}\left(t_{i}\right)+\sum_{j=1}^{3}\left(\Phi_{sq}\left(x_{j}\left(t_{i}\right),y_{j}\left(t_{i}\right)\right)\times \left(GRF_{whj}\left(t_{i}\right)+\;IF_{whj}\left(t_{i}\right)\right)\right)+\sum_{j=1}^{3}\left(\Phi_{sq}\left(x_{j}\left(t_{i}\right),y_{j}\left(t_{i}\right)\right)\times IF_{sihj}\left(t_{i}\right)\right)$$
IX. The response of each mode of the structure was then calculated using the Newmark integration method [30], using the displacement and velocity of the previous timestep as initial conditions for the current timestep:
  (12) $$m_{sq}{\ddot{x}}_{sq}\left(t_{i}\right)+c_{sq}{\dot{x}}_{sq}\left(t_{i}\right)+k_{sq}x_{sq}\left(t_{i}\right)=F_{wh+sih+sh\to sq}\left(t_{i}\right)$$
X. The process was repeated.

### 4.3. Vibration Serviceability Assessment

The agent-based response simulation process provides the time history of the acceleration levels experienced by each occupant/pedestrian *‘j’* ${\ddot{z}}_{s\to hj}\left(t\right)$ based on their location (continuously changing for walking subjects) on the structure (Equation (7)). These time histories ${\ddot{z}}_{s\to hj}\left(t\right)$ are then connected together in series to form the time history of the vibration experience of all the occupants ${\ddot{z}}_{s\to h}\left(t\right)$. This method takes into account both the actual level of vibrations each pedestrian experienced based on their (potentially) moving location on the structure and the duration each occupant was exposed to a certain level of vibration.

The CDF of the total vibration experience time history ${\ddot{z}}_{s\to h}\left(t\right)$, termed the Moving-Location Cumulative Distribution Function (ML CDF) by Shahabpoor et al. [25], is then calculated to assess the vibration serviceability of the structure. For any particular amplitude of ${\ddot{z}}_{s\to h}\left(t\right)$, ML CDF ordinate provides the probability that a pedestrian does not experience a vibration level higher than the selected amplitude.

## 5. Experimental Verification

In this section, the performance of the proposed AVSA in simulating HSEI, estimating the occupied structure’s modal properties, and the ML CDF of the vibration experience of the occupants as the ‘receiver’ of vibrations are assessed.

In the first step, the ABM was used to simulate FRF tests (Tests 3–6—Table 1). Only the inputs available at the design stage were used in simulations: room layout, number of walking and sitting subjects, and the location of sitting subjects. The shaker force was also considered known.

**Figure 9:**
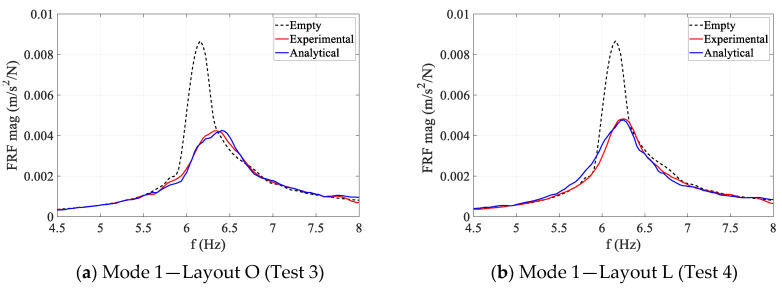

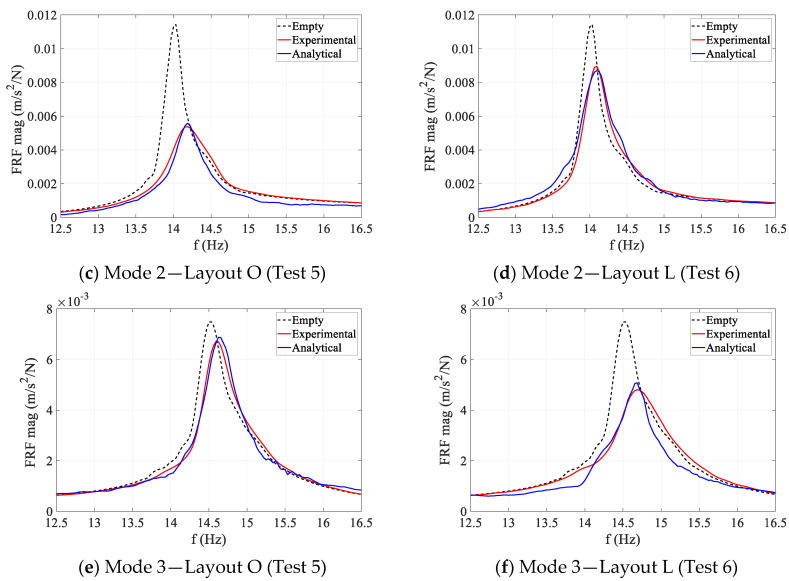
Comparison of the occupied structure experimental and analytical FRFs.

For each test, the physical acceleration response of the structure at the anti-node of the corresponding mode was calculated. This simulated response and the corresponding shaker force were then used to calculate the accelerance FRF of the occupied structure and were compared with their experimental counterpart in Figure 9. As can be seen in this figure, the analytical FRFs consistently match their experimental counterparts with high accuracy. The maximum error in the estimated modal frequency and damping ratio of the occupied structure were 0.7% and 4.5%, respectively, across all three modes. This indicates that AVSA has been successful in simulating both HSI mechanisms and human movement patterns on the floor realistically.

**Figure 10:**
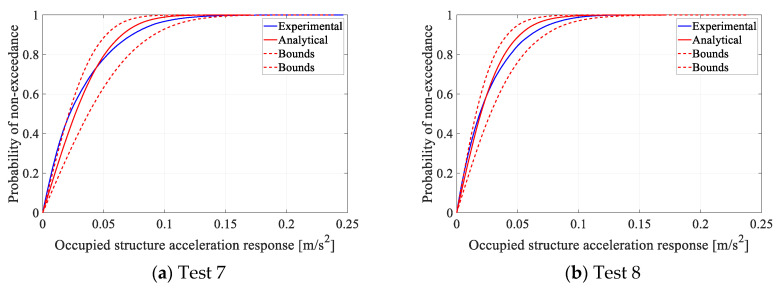
Comparison of experimental and analytical ML CDFs.

In the second step, AVSA was used to simulate Tests 7 and 8 (Table 1) to calculate the time history of the acceleration levels experienced by each occupant ‘*j*’ ${\ddot{z}}_{s\to hj}\left(t\right)$. The acceleration measured by three accelerometers (TP1 to TP16—Figure 1) closest to each occupant in each timestep were triangulated (linearly based on the distance of the subject to the sensors) to calculate the experimental acceleration time history experienced by each occupant/pedestrian. The ML CDF of both analytical and experimental acceleration time histories experienced by all occupants are then calculated and compared in Figure 10.

To estimate 95% confidence intervals for the analytical ML CDF, an equivalent to 15 h of structural response was simulated for each test to ensure the standard error σ values of the mean a_95%_, a_85%_, a_75%_, and a_50%_ are below 5% of their mean values (for more details see [25]). The population is then defined as the full length of this simulated response with σ < 0.05µ, and the sample is defined as a randomly selected block (window) of the response with a duration of 240 s, equal to the corresponding measured response. For Tests 7 and 8, all sample data blocks with a maximum 95% overlap were drawn from the 15 h simulated response. The CDF of each of these sample data blocks was calculated. For each response value on the horizontal axis of the ML CDF, the confidence interval [μ−2σ,μ+2σ] is calculated using the corresponding values on all sample CDFs. These confidence intervals/bounds (Figure 10—two dashed red curves) represent the corresponding lower and upper confidence limits/bounds of the sample CDF curves. Conceptually, this means that for any arbitrary 240 s response measured on the test structure, the measured structural response CDF will be between the lower and upper confidence bounds with 95% probability (assuming normal distribution of data points).

As can be seen in Figure 10, the analytical ML CDF approximates the experimental ML CDF with high accuracy for both Tests 7 and 8, and the experimental ML CDF is well within the 95% confidence limits estimated by AVSA.

## 6. Conclusions

This study proposes a generic Agent-based Vibration Response Simulation and Serviceability Assessment framework (AVSA), allowing for cross-modality simulation of the interactions, simultaneous simulation of multiple modes of structure, effortless and expandable simulation of human-environment interactions for any interior layout and usage pattern, simultaneous simulation of a combination of different human postures and tasks, and autonomous switching between the tasks within the simulations. Application of AVSA on a real-life floor structure with closely spaced modes of vibration and a combination of sitting and walking human subjects showed a consistent and accurate performance of the framework with a maximum error of 0.7% in estimated modal frequency and 4.5% for damping ratio of the occupied structure. The CDF of the acceleration response experienced by the occupants was within the 95% confidence interval predicted by the AVSA framework.

The key advantages of the proposed AVSA framework are its flexibility, effortless adaptability, and limitless expandability to simultaneously simulate different human movement patterns, interaction modalities, human tasks and postures, and multiple modes of a structure. The model can be extended easily to simulate the effects of non-structural elements such as partitions by simulating them as a mass-spring-damper system and adding them to the ABM as agents.

Due to the limited number of inputs required and the versatility of the ABM in adding and removing different agents (e.g., different human postures/tasks, non-structural elements, modes of structure, etc.), the proposed AVSA method is an easy-to-use standalone application that can be used by design engineers at the design stage.

The proposed framework is validated against one dataset in this study. Further application of this framework to other structural systems and usage patterns would be needed to further validate its performance.

## Figures and Tables

**Table 1:** Experimental modal properties of the structure.

ID	Description	Mode	Layout	f(Hz)	ζ(%)	m(kg)	Δf(%)	Shape
01	FRF—Empty	1	-	6.15	1.8	3000		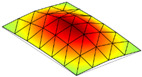
03	FRF—3W-3S	1	O	6.32	3.6	3175	2.8%	
04	FRF—3W-3S	1	L	6.24	3.2	3125	1.5%	
02	FRF—Empty	2	-	14.02	0.78	5600		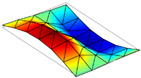
05	FRF—3W-3S	2	O	14.18	1.60	5700	1.1%	
06	FRF—3W-3S	2	L	14.10	0.91	5800	0.6%	
02	FRF—Empty	3	-	14.52	1.15	5600		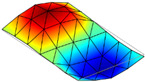
05	FRF—3W-3S	3	O	14.60	1.27	5700	0.6%	
06	FRF—3W-3S	3	L	14.69	1.78	5800	1.2%	
07	RM—3W-3S	All	O	-	-	-		-
08	RM—3W-3S	All	L	-	-	-		-

Notes: FRF: Frequency Response Function measurement; RM: response monitoring test; f: modal frequency; ζ: modal damping ratio; m: modal mass; Δf: change in modal frequency; W: walking; S: sitting.

## Data Availability

The available data are contained within the article.

## Nomenclature

Symbol/acronym	Description
HSI	Human-Structure Interactions
HEI	Human-Environment Interactions
HHI	Human-Human Interactions
HSEI	Human-Structure-Environment Interactions
VSA	Vibration Serviceability Assessment
I-VSA	Interaction-based Vibration Serviceability Assessment method
AVSA	Agent-based Vibration Serviceability Assessment method
FRF	Frequency Response Function
CDF	Cumulative Distribution Function
ML CDF	Moving-Location Cumulative Distribution Function
RM	Response monitoring test
TP	Test point
ABM	Agent-Based Model
SDOF	Single degree of freedom
DoF	Degree of freedom
GRF	Ground reaction force (walking)
SFM	Social Force Model
M	Mass
C	Damping coefficient
K	Stiffness
wh	Walking human
sih	Sitting human
IF	Interaction force
t	Time
x(t),y(t)	[x,y] Coordinates of each person on the floor at time *t*
V	Walking speed, heading velocity
$F_{Res}$	Resultant force (SFM)
$F_{M}$	Motive force (SFM)
$F_{IP}$	Inter-pedestrian force (SFM)
$F_{B}$	Boundary force (SFM)
$t_{r}$	Agent reaction time (SFM)
$m_{p}$	Mass constant (SFM)
$k_{1}$	Force constant (SFM)
$k_{2}$	Distance constant (SFM)
$r_{IP}$	Agent personal radius in inter-personal interaction (SFM)
$r_{B}$	Agent personal radius in relation to boundaries (SFM)
$d_{E}$	Distance of the agent from the edge (SFM)
$d_{C}$	Distance of the agent from the corner (SFM)
c	Field of view reduction factor (SFM)
∅	Unity-normalized mode shape magnitude
$t_{sim}$	Total time all subjects have spent in each of the mode-shape zones during each test (simulation)
$t_{exp}$	Total time all subjects have spent in each of the mode-shape zones during each test (experimental)
Err	Error value
f	Modal frequency
ζ	Modal damping ratio
m	Modal mass
Δf	Change in modal frequency
W	Walking
S	Sitting
$\ddot{z}$	Acceleration in the vertical direction
$F_{sh}$	Shaker force
sq	Mode ‘q’ of structure
μ	Mean value
σ	Standard deviation
